# Describing the Clinical and Laboratory Features and HLA-B Pattern of Adult-Onset Idiopathic Autoimmune Uveitis at a Tertiary Hospital in South India: A Cross-Sectional Study

**DOI:** 10.1155/2022/5032881

**Published:** 2022-02-08

**Authors:** Jyothi Visalakshy, Sandeep Surendran, Salil Ganu, Kannisha Shah, C. B. Mithun, Vishal Marwaha, Lalitha Biswas, Niveditha Kartha, Gopal Pillai

**Affiliations:** ^1^Department of Rheumatology, Amrita Institute of Medical Sciences, Amrita Vishwa Vidyapeetham, Kochi, Kerala, India; ^2^Department of Ophthalmology, Amrita Institute of Medical Sciences, Amrita Vishwa Vidyapeetham, Kochi, Kerala, India; ^3^Department of Molecular Biology, Amrita Institute of Medical Sciences, Amrita Vishwa Vidyapeetham, Kochi, Kerala, India; ^4^Department of Biostatistics, Amrita Institute of Medical Sciences, Amrita Vishwa Vidyapeetham, Kochi, Kerala, India

## Abstract

**Introduction:**

There is a scarcity of information available on clinical and laboratory features of adult-onset idiopathic autoimmune uveitis. Therefore, we conducted a single centre descriptive cross-sectional study. *Patients and Methods*. A chart review of all patients with idiopathic autoimmune uveitis with onset after 18 years of age who were referred to the rheumatology department between January 2017 and December 2018 was performed. Their clinical features, demographic features, and HLA-B genotypes were documented and described.

**Results:**

Out of 210 patients referred to rheumatology, 66 were found to have uveitis, and 16 of these had an adult-onset idiopathic autoimmune uveitis. Apart from a slight female preponderance (62.5%), our patients were characterized by a high proportion of panuveitis (4 out of 16, i.e., 25%). There was an increased frequency of occurrence of synechiae (5 out of 16, i.e., 31.3%), retinal vasculitis (4 out of 16, i.e., 25%), optic disc edema (3 out of 16, i.e., 18.8%), and cystoid macular edema (seen in 2 patients, i.e., 12.5%). These features correlated with the anatomical subtypes. Retinal vasculitis and optic disc edema present in three fourth of all panuveitis cases were the most prominent features. The odds of finding HLA-B∗35 in retinal vasculitis were 33 times higher than odds of finding it in idiopathic autoimmune uveitis patients not having retinal vasculitis (OR 33; 95% CI 1.6–698).

**Conclusion:**

Idiopathic autoimmune uveitis in our patients is characterized by a high frequency of panuveitis and retinal vasculitis, and complications with a probable association between HLA-B∗35 and retinal vasculitis.

## 1. Introduction

Uveitis is an important cause of ocular morbidity throughout the world leading to 5–10% of all causing visual impairment [1]. About one third of all cases of uveitis result in a significant visual loss [2]. Nearly 17–52 new cases of uveitis per 100,000 population occur in the world with a prevalence of 38–714 cases per 100,000 population [3]. India alone has a prevalence of 730 uveitis cases per 100,000 population, and a tertiary care center in India reported an incidence of 1.5% [4, 5]. There is a regional difference documented in different parts of India. The common complications of uveitis include cataract, glaucoma, band keratopathy, cystoid macular edema, optic disc edema, vitreal hemorrhages, epiretinal membrane, and retinal neovascularization [6–10]. Retinal vasculitis is seen accompanying a severe form of posterior uveal inflammation. Despite the burden of this problem, the classification of uveitis has always been an ever-evolving process due to the discovery of new diagnostic modalities. There are various ways in which uveitis has been traditionally classified. These include anatomical classification, classification based on chronicity, and etiological classification. Most recent etiological classification schemes classify uveitis into infectious and noninfectious varieties. The noninfectious uveitis is further classified into malignancy-related and autoimmune varieties. The subsequent classification of autoimmune uveitis is performed on the basis of its association with systemic autoimmune disease as a systemic disease-associated autoimmune uveitis and idiopathic autoimmune uveitis [11]. An Indian study from 1996 reports that the etiology of uveitis is established in 59.31% of the cases [4]. The percentage of which is likely to be increasing in subsequent decades. Although there has been ample literature describing the burden of noninfectious uveitis and its complications, the literature addressing the same in autoimmune uveitis as a group has been scarce. The lack of availability of the literature pertaining to prevalence, natural history, complications, and sequelae is particularly remarkable for idiopathic-autoimmune variety of autoimmune uveitis. In our clinical experience, we found that the idiopathic autoimmune uveitis has a fairly distinct clinicolaboratory phenotype with a high frequency of various complications and the associated retinal vasculitis. Therefore, we have tried to describe our experience with adult-onset idiopathic autoimmune uveitis and its complications with a special reference to retinal vasculitis.

### 1.1. Objective

The objective of this study is to describe the clinical and laboratory features of an idiopathic autoimmune uveitis.

## 2. Patients and Methods

### 2.1. Study Design and Patient Selection

A screening of the electronic medical hospital records was done for all patients who consulted the rheumatology department of a tertiary care hospital in south India between January 2017 and December 2018. Among the screened charts, the patients diagnosed to have uveitis by an expert ophthalmologist were selected. Uveitis was considered to be idiopathic autoimmune uveitis if the following criteria were fulfilled: (1) all known causes of infectious uveitis had been ruled out; (2) the systemic autoimmune diseases such as sarcoidosis, Behcet's disease, spondyloarthropathy, or connective tissue disorder syndromes (SLE, RA, and Sjogren's syndrome) were excluded by a rheumatologist on clinical grounds as well as with respective classification criteria (enlisted in,Table 1); (3) a favorable expert opinion from an ophthalmologist for the diagnosis of idiopathic autoimmune uveitis was taken; and (4) malignancies were ruled out whenever suspected patients were selected if the onset of their first uveitis symptom happened after 18 years of age. All patients who were residents of the state of Kerala were included because it is a tourist center. All patients with idiopathic autoimmune uveitis were treated in a multidisciplinary setting as a part of the institutional standard of care, and investigations for determining the cause of the uveitis were carried out in all patients in a tailored approach [12, 13]. All the idiopathic autoimmune uveitis patients who were diagnosed in such a manner were eligible for enrollment after confirming verbal (telephonic) informed consent. Patients were enrolled in the study if adequate details of their clinical features and treatment were available. Recruited patients were further classified using the Standardization of Uveitis Nomenclature (SUN) Working Group of anatomical classes of uveitis [14]. The characteristic features and treatments received by idiopathic autoimmune uveitis patients in general and in their anatomical subclasses were described.

### 2.2. Clinical Details and Outcome Measures

Basic demographic details (age and gender) were noted. Duration of disease and past treatment history including the response to previous treatment were documented. Uveitis was classified according to anatomical localization as anterior (iritis, iridocyclitis, and anterior cyclitis), intermediate (pars planitis), posterior (focal, multifocal, or diffuse choroiditis, chorioretinitis, retinochoroiditis, and neuroretinitis), or panuveitis (inflammation of the anterior chamber, vitreous, and retina or choroid). In addition, information about acute, chronic, or recurrent nature and laterality (unilateral Vs bilateral) of uveitis was also collected. Extraocular features were also documented. Examination findings such as the presence of synechiae, vasculitis, retinal hemorrhages, hypopyon, cataract, epiretinal membranes, and features suggestive of glaucoma were also noted. The baseline CRP (mg/L) and ESR (mm/Hr.) levels and the nature of treatment received (topical/local, oral, or parenteral steroids, DMARD initiation, etc.,) were documented. Change in visual acuity was documented whenever data were available.

### 2.3. HLA-B Genotype Analysis Procedure

Genomic DNA is extracted from the peripheral blood samples using the salting-out method. The DNA obtained is quantified using the spectrophotometer (biophotometer, Germany) and adjusted to a concentration of 30 ng/µl. HLA-B tissue typing is performed using the HLA-B sequence-specific primer-based low-resolution kit (BAG Healthcare, Germany) according to the manufacturer's protocol. The PCR products are electrophoresed in a 3% agarose gel. HLA alleles are assigned based on the band pattern using the HistoMatch software (BAG Healthcare, Germany).

### 2.4. Statistical Methods

The analysis was carried out using SPSS25 software. Descriptive statistics were summarized using means and percentages.

## 3. Results

**Figure 1:**
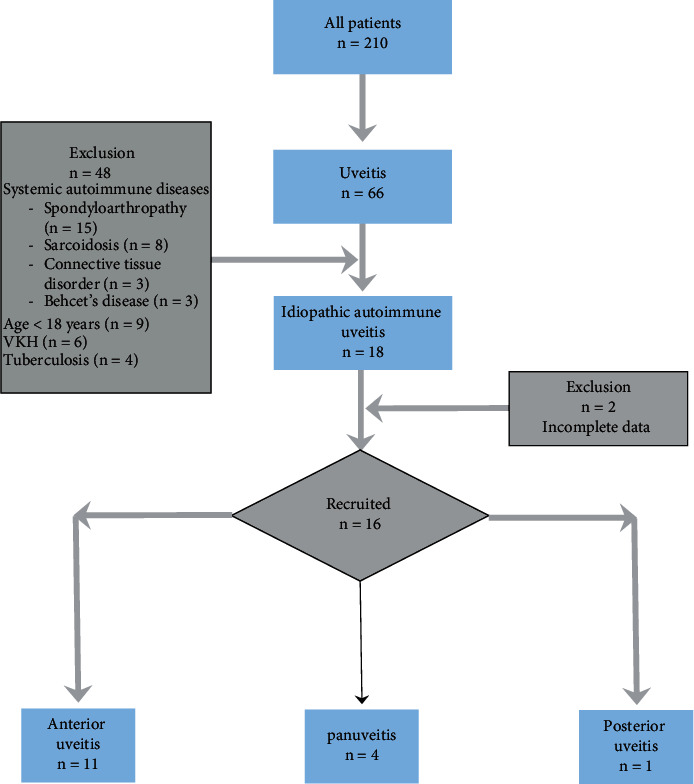
Patient flow in the study.

Charts of a total of 210 patients who were referred to the rheumatology department for the evaluation of autoimmune uveitis were screened. Total 66 patients were found to have a diagnosis of uveitis, out of which 48 patients were excluded, and 18 patients were found to have a diagnosis of idiopathic autoimmune uveitis. Two patients who did not have a complete clinical record were excluded. A total of 16 patients with a diagnosis of idiopathic uveitis were selected. All patients included in this study had recognized themselves as ethnical south Indians.  Figure 1 summarizes the patient flow in the study. The baseline demographic characteristics and disease profile of all patients are described in Table 2. The median age of autoimmune idiopathic uveitis patients was found to be 41 (37–58.2) years. There was a female preponderance among idiopathic autoimmune uveitis cases, and the median disease duration was 24 (6.3–36) months. Eleven patients had anterior uveitis, 4 had panuveitis, and 1patient had posterior uveitis. At the time of their first visit, the previous treatment record was available for 13 patients, out of which 3 patients were treatment naïve, 8 had received some form of treatment including 4 patients each being treatment refractory and treatment responsive. Refractoriness was defined as no inactivity or reactivation of uveitis at doses of topical steroid of >3 drops per day given for at least 12 weeks. [15]The diagnosis was confirmed in all patients as mentioned in the methodology section. Three patients including two having anterior uveitis and one having panuveitis were found to have a tuberculin sensitivity test of more than 5 cm but less than 10 cm. These did not have characteristic pulmonary or extrapulmonary features of TB. These also did not have specific ocular features. As these patients had a history of BCG vaccination in childhood and tested negative on interferon gamma release assay, they were considered to have inadequate evidence to be classified as having an ocular TB although tissue despite having no tissue-culture testing. Consent for the same was refused by the patient. One patient among these did receive empirical antitubercular treatment for latent treatment in anticipation of future need for a TNF-alfa inhibitor.

Among all idiopathic autoimmune uveitis patients, at the time of patient recruitment in the study, a large majority of our patients had received topical steroids and topical cycloplegics. Systemic steroids, including some form of intravenous steroid in selected patients, were used in a total of 9 patients. Almost half of our patients had received at least one conventional synthetic DMARD (cs DMARD), whereas three had required a second cs DMARD. None of our patients had required a biological DMARD (b DMARD).

### 3.1. Clinical and Laboratory Features of Idiopathic Autoimmune Uveitis

The clinical and laboratory characteristics of patients are described in Table 3. A slightly above half of our patients had unilateral uveitis. Pain and redness of eyes were the most common symptoms, whereas synechiae formation was the most common complication encountered. A sizeable number of patients had blurring of vision. Among the three patients who had a cataract, one was found to have a probable steroid-induced cataract (with a history of long duration of steroid use), and another one had a probable senile (nuclear sclerosing) cataract. Hypopyon, epiretinal membranes, or glaucoma were not found in any of these patients. Almost one third of these patients had retinal vasculitis most of whom also had a cystoid macular edema and/or optic disc edema. Majority of these patients were found to have panuveitis, and one had posterior uveitis. However, none of these patients had vitreous hemorrhages or retinal neovascularization. Visual acuity data for first and last hospital visits were available for only 8 patients. Moreover, many of these patients were initiated on treatment for uveitis long before their first visit. Since their first visit, visual acuity had improved, remained status quo, and worsened in 3 patients each.

**Figure 2:**
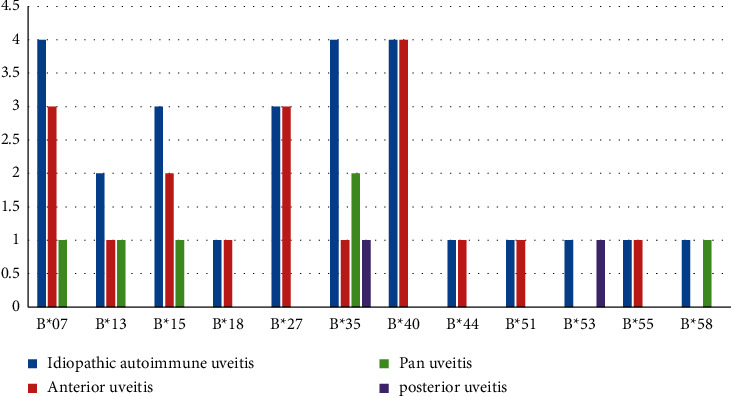
HLA-B typing.

A total of nine patients had at least one extraocular feature, and two had more than one extraocular feature. Inflammatory low backache was present in six patients, joint pain was present in four patients, and one patient had heel pain. Joint pain and heel pain are mechanical in nature. None of these patients with inflammatory low backache had sufficient features to be classified as a spondyloarthropathy as per ASAS criteria. Markers of systemic inflammation were raised in about one third of patients at the time of their first visit. CRP and ESR were raised in 6 patients each. HLA-B type distribution is denoted in Figure 2. HLA-B∗07, B∗35, and B∗40 were present in 4 patients each. HLA-B∗35 was seen in 3 patients who had posterior segment involvement. Two patients were found to have HLA-B∗51. However, they did not have adequate clinical features to be classified as Behcet's disease according to ICBD criteria.

### 3.2. Clinical and Laboratory Characteristics of Retinal Vasculitis

A sizeable number of patients in this study were found to have retinal vasculitis. Therefore, the clinical and laboratory features of retinal vasculitis are described in Table 4. There were 4 retinal vasculitis patients in this study out of which 3 had panuveitis, and one had posterior uveitis. The clinical features represented the anatomical type of uveitis; therefore, blurring of vision followed by pain and redness of eyes were the most common symptoms noticed. Optic disc edema was the most common complication apart from optic neuritis, cataract, and cystoid macular edema present in one patient each. One patient each had inflammatory low backache and mechanical heel pain. It was also noticed that HLA-B∗35 was present in 3 patients with retinal vasculitis, indicating that there are 33 times higher odds of having the presence of HLA-B∗35 among patients with retinal vasculitis as compared to autoimmune idiopathic uveitis patients not having retinal vasculitis (OR 33; 95% CI 1.6–698).

## 4. Discussion

Indian studies report the prevalence rate for endogenous uveitis as 310/100,000 population with posterior uveitis involvement as a major risk factor for blindness [16]. Indian cohort studies also report etiologies of uveitis, and studies from both north and south India report that idiopathic uveitis accounts for 15–20% of patients presenting to tertiary ophthalmological clinics [17, 18]. The literature review shows that a classification scheme similar to ours classifying the uveitis into an idiopathic autoimmune variety has been carried out previously by Prete et al. [11]. In this tertiary center-based retrospective analysis of 104 patients from Italy, a comparative description of idiopathic autoimmune uveitis with systemic disease-associated autoimmune uveitis has been given.

Our study was characterized by the predominance of anterior uveitis (68.8%) and panuveitis (25%) as compared to posterior uveitis (6.3%). This was different from that found in a larger study conducted in the south Indian population having uveitis by Biswas et al. [4]. In this study, they found that anterior uveitis was found in 40%, posterior uveitis in 29%, intermediate uveitis in 17%, and panuveitis in 14% of all patients. In the study by Prete et al., anterior uveitis was seen in 42.7% patients, posterior uveitis in 46.7%, intermediate uveitis in 6.7%, and panuveitis in 10.3% patients. Another large systematic review by Barisani-Asenbauer et al. also described the prevalence of panuveitis in the range of 2.38% [19]. This pattern of the higher percentage of anterior uveitis and panuveitis cases was reflected in the frequency of symptoms and complications of uveitis in our study. Consistent with findings in the study conducted by Prete et al., the redness of eyes, pain, and blurring of vision were the predominant complaints in our patients.

In our study, it was found that patients having anterior uveitis had clinical features similar to spondyloarthropathy-related typical (HLA-B∗27 associated) acute anterior uveitis, such as acute presentation with a unilateral or alternating bilateral pattern, recurrent nature, association with HLA B27, and presence of inflammatory low backache [20, 21]. An odd feature noted was the female preponderance in patients having a typical acute anterior uveitis. We found that there is a mild female preponderance in our study. Another Indian study in patients with uveitis indicates male preponderance worldwide although it is considered to be having an equal distribution [16]. A retrospective study from Holland showed that about 53% panuveitis, 41% of intermediate uveitis, and 28% posterior uveitis had cystoid macular edema [22]. We found that it was present in 12.5% of all uveitis patients and 75% of patients having panuveitis. The prevalence of cataract, which is another major complication, is reported to be in 18–35% of all uveitis cases [23]. This was comparable to our study where 18.8% patients developed cataract. The prevalence of retinal vasculitis among uveitis cases was found to be 14.9% in a previous study [24]. This is much lower than in our patients with idiopathic autoimmune uveitis. The higher proportion of retinal vasculitis was probably attributable to the presence of a higher number of patients with a posterior segment involvement. Overall, the percentage of patients who developed various complications of uveitis in our patients was also much higher than that described in the literature [20].

The HLA-B∗35 was the most frequent HLA-B serotype present in panuveitis and posterior uveitis patients. HLA-B∗35 is one of the biggest HLA-B serotype groups in existence. There are currently 86 polypeptide isoforms and 97 nucleotide variants present in this group [25]. Apart from its well-established association with the accelerated progression of HIV infection to AIDS, HLA-B∗35 has been reported to be associated with Juvenile idiopathic arthritis-related uveitis, self-limiting unclassified rheumatism, sacroiliitis in undifferentiated spondyloarthropathy, and oral aphthosis [26–32]. The literature on ocular involvement in HLA-B∗35 positivity is scarce and shows its presence in up to 24% of uveitis patients in a small-sized prospective study [33]. We did not find any previous report of the association of retinal vasculitis with HLA-B∗35. HLA-B∗35 is responsible for endoplasmic reticulum stress response by upregulation of endothelin 1 and downregulation of nitric oxide in endothelial cells of pulmonary vasculature [34]. This causes endothelial cell dysfunction and subsequent pulmonary vasoconstriction. Therefore, whether a similar process of endothelial stress and unfolded protein response associated with HLA∗B35 causes inflammation of retinal blood vessels in posterior uveitis remains to be seen. Another mechanism which can be considered plausible is like arthritogenic peptide hypothesis in spondyloarthropathy. Evidence for this comes from the seminal study conducted by Petty et al. on JRA children. In this study, they found that the JRA children with uveitis who had the presence of HLA-B∗35 had a higher frequency of antibody to soluble retinal antigen. [27] This might be a result of a process in which HLA-B∗35 shows a preferential binding to soluble retinal antigens and their subsequent presentation to lymphocytes causing an abnormal immune response against them like that seen in the case of HLA-B∗27 and arthritogenic peptides [35].

There are two main limitations of our study, namely (1) a small number of patients and (2) the possibility of a referral bias. Although this limits the generalizability of our study results, it lays the foundation for generating a hypothesis that adult-onset idiopathic autoimmune uveitis is characterized by a higher percentage of panuveitis and its complications, particularly with retinal vasculitis. It also elucidates the increased odds of the presence of HLA-B∗35 among patients with retinal vasculitis. These conclusions can be tested in a prospective cohort study.

## 5. Conclusion

Adult-onset idiopathic autoimmune uveitis in our patients from southern India is characterized by the high frequency of panuveitis, retinal vasculitis, and complications. Also, there are higher odds of having the presence of HLA-B∗35 in patients having retinal vasculitis.

## Figures and Tables

**Table 1:** Classification criteria used for systemic autoimmune diseases associated with uveitis.

Disease	Classification or diagnostic criteria
Spondyloarthropathy	ASAS
Psoriatic Arthritis	CASPER
Systemic Lupus Erythematosus	SLICC2012
Behcet's disease	ICBD2006
Rheumatoid arthritis	ACR2010
ANCA-associated vasculitis	ACR1990
Sarcoidosis	Clinical diagnosis confirmed by histological evidence

**Table 2:** Baseline characteristics.

Features	Idiopathic autoimmune uveitis (*N* = 16)	Anterior uveitis (*N* = 11)	Panuveitis (*N* = 4)	Posterior uveitis (*N* = 1)
Age in years, median (IQR)	41 (37–58.2)	41 (40–60)	41 (23.8–65)	37
Female, *n* (%)	10 (62.5)	6 (54.5)	4 (100)	—
Uveitis duration in month, median (IQR)	24 (6.3–36)	12 (6–36)	30 (11.3–126)	36

Past treatment history				
Treatment refractory, *n*' (*n*)	4 (13)	2 (8)	2 (4)	1
Treatment naïve, *n*' (*n*)	5 (13)	3 (8)	1 (4)	—
Treatment responsive, *n*' (*n*)	4 (13)	3 (8)	1 (4)	—

Tuberculin sensitivity test, n' (n)				
Interferon gamma release assay, *n*' (*n*)	3 (12)	2 (7)	1 (3)	0 (1)
CXR PA view for TB, *n*' (*n*)	0 (7)	0 (3)	0 (1)	0 (1)
Sacroiliac MRI, *n*' (*n*)	0 (12)	0 (9)	0 (2)	0 (1)
ANA (IFA), *n*' (*n*)	0 (6)	0 (5)	—	0 (1)
AntiPR3/MPO, *n*' (*n*)	2 (5)	1 (3)	1 (1)	—
ACE, *n*' (*n*)	0 (5)	0 (3)	0 (1)	0 (1)
CRP, median (IQR)	0 (12)	0 (7)	0 (4)	0 (1)
ESR, median (IQR)	5.1 (2–8)	5.1 (2.4–8)	7.4 (2–32)	1.1
Topical treatment	16 (10–40)	18 (10–40)	16 (15–57)	6
Topical steroid, *n* (%)	15 (93.8)	11 (100)	4 (100)	—
Topical cycloplegics/mydriatics, *n* (%)	12 (75)	9 (75)	3 (75)	—

Regional therapy				
Intraocular steroid, *n* (%)	1 (6.3)	—	1 (25)	—
Systemic steroids *n* (%)	9 (56.3)	4 (36.4)	3 (75)	—
Intravenous steroid, *n* (%)	4 (25)	2 (18.2)	1 (25)	—
Oral steroid, *n* (%)	8 (50)	3 (27.3)	3 (75)	11

Additional immunosuppression				
Steroid sparing, *n* (%)	7 (43.8)	5 (45.5)	2 (50)	1
Mycophenolate mofetil, *n* (%)	2 (12.5)	—	2 (50)	1
Methotrexate, *n* (%)	4 (25)	3 (27.3)	1 (25)	—
Sulfasalazine, *n* (%)	3 (18.8)	3 (27.3)	—	—
Other therapy, *n* (%)	1 (6.3)	—	1 (25)	—
2^nd^ DMARD, *n* (%)	3 (18.8)	2 (18.2)	1 (25)	—

*n*' = frequency of positive results, *n* = total number of patients with a variable, *N* = total number of patients in the group.

**Table 3:** Clinical and laboratory features of idiopathic autoimmune uveitis.

Features	Idiopathic autoimmune uveitis (*N* = 16)	Anterior uveitis (*N* = 11)	Panuveitis (*N* = 4)	Posterior uveitis (*N* = 1)
Unilateral	9 (56.3)	5 (45.5)	3 (75)	1

Symptoms				
Pain in eye, *n* (%)	12 (75)	10 (91)	2 (50)	—
Redness of eye, *n* (%)	12 (75)	11 (100)	1 (25)	—
Epiphora, *n* (%)	4 (25)	4 (36.4)	—	—
Photophobia, *n* (%)	7 (43.8)	4 (63.6)	—	−1
Blurring of vision *n* (%)	6 (37.5)	1 (9.1)	4 (100)	—
Others, *n* (%)	1 (6.3)	—	1 (25)	

Examination findings				
Synechiae, *n* (%)	5 (31.3)	4 (36.4)	1 (25)	—
Cataracts, *n* (%)	3 (18.8)	1 (9.1)	2 (50)	—
Retinal vasculitis, *n* (%)	4 (25)	—	3 (75)	1
Cystoid macular edema, *n* (%)	2 (12.5)	—	2 (50)	—
Optic disc edema, *n* (%)	3 (18.8)	—	3 (75)	—
Optic neuritis, *n* (%)	1 (6.3)	—	1 (25)	—

Visual Acuity				
Improved, n'(*n*)	3 (9)	1 (7)	2 (2)	—
Status quo n'(*n*)	3 (9)	3 (7)	—	
Worsened, n'(*n*)	3 (9)	3 (7)	—	

Extraocular features, *n* (%)	9 (56.25)	8 (72.7)	1 (25)	1
Inflammatory LBA, *n* (%)	6 (37.5)	5 (45.5)	—	1
Joint pain, *n* (%)	4 (25)	4 (36.4)	—	—
Heel pain *n* (%)	1 (6.3)	1 (9.1)	—	—
More than 1 feature	2 (12.5)	2 (18.2)	—	—

Inflammatory markers				
Raised CRP, *n* (%)	6 (37.5)	4 (36.4)	2 (50)	1.1
Raised ESR, *n* (%)	6 (37.5)	5 (45.5)	1 (25)	6

(a) *n*' = frequency of positive results, *n* = total number of patients with a variable, *N* = total number of patients in the group. (b) CRP >6 mg/L. (c) ESR >22 mm/Hr.

**Table 4:** Clinical and laboratory features of retinal vasculitis.

Features	Retinal vasculitis (*N* = 4)	No retinal vasculitis (*N*' = 12)
Age in years, median (IQR)	33 (23.8–49)	45 (40–60)
Female gender, *n* (%)	3 (75)	7 (58.3)
Uveitis duration, median (IQR)	36 (27–126)	12 (6–34)
Uveitis laterality unilateral, *n* (%)	4 (100)	5 (41.7)

Uveitis type		
Posterior uveitis, *n* (%)	1 (25)	—
Panuveitis, *n* (%)	3 (75)	1 (8.3)

Past treatment history		
Treatment refractory, *n*' (*n*)	1 (4)	3 (20)
Treatment naïve, *n*' (*n*)	2 (4)	3 (10)
Treatment responsive, *n*' (*n*)	1 (4)	3 (40)

Symptoms		
Pain, *n* (%)	2 (50)	10 (83.3)
Redness, *n* (%)	1 (25)	11 (91.7)
Epiphora, *n* (%)	-	4 (33.3)
Photophobia, *n* (%)	—	7 (58.3)
Blurring of vision, *n* (%)	4 (100)	2 (16.7)
Black spots, *n* (%)	1 (25)	—

Examination findings		
Synechiae, *n* (%)	—	5 (41.7)
Cataract, *n* (%)	1 (25)	2 (16.7)
Cystoid macular oedema, *n* (%)	1 (25)	1 (6.3)
Optic disc oedema, *n* (%)	2 (50)	1 (6.3)
Vitreous hemorrhages, *n* (%)	—	—
Optic neuritis, *n* (%)	1 (25)	—

Extraocular features		
Inflammatory low backache	1 (25)	5 (41.7)
Heel pain	1 (25)	1 (6.3)
Joint pain	—	4 (33.3)
More than 1 feature	—	2 (16.7)

Inflammatory markers		
CRP, median (IQR)	1.6 (1.1–32)	5.4 (2.6–7.9)
ESR, median (IQR)	15.5 (8.3–46.8)	18 (10–40)

HLA-B patterns		
B07, *n*	0	4
B13, *n*	1	1
B15, *n*	0	3
B18, *n*	0	1
B27, *n*	0	3
B35, *n*	3	1
B40, *n*	0	4
B44, *n*	0	1
B51, *n*	0	2
B52, *n*	0	0
B53, *n*	1	0
B55, *n*	0	1
B58, *n*	1	0

*n*' = frequency of positive results, *n* = total number of patients with a variable, *N* = total number of patients in the group 3.

## Data Availability

Data used in this study are available from the corresponding author upon request.
